# Supplementary figures and images for: A synthetic cell density signal can drive proliferation in chick embryonic tendon cells and tendon cells from a full size rooster can produce high levels of procollagen in cell culture
Source: PeerJ. 2022 Dec 12;10:e14533. doi: 10.7717/peerj.14533 (PMC9753744; doi:10.7717/peerj.14533)

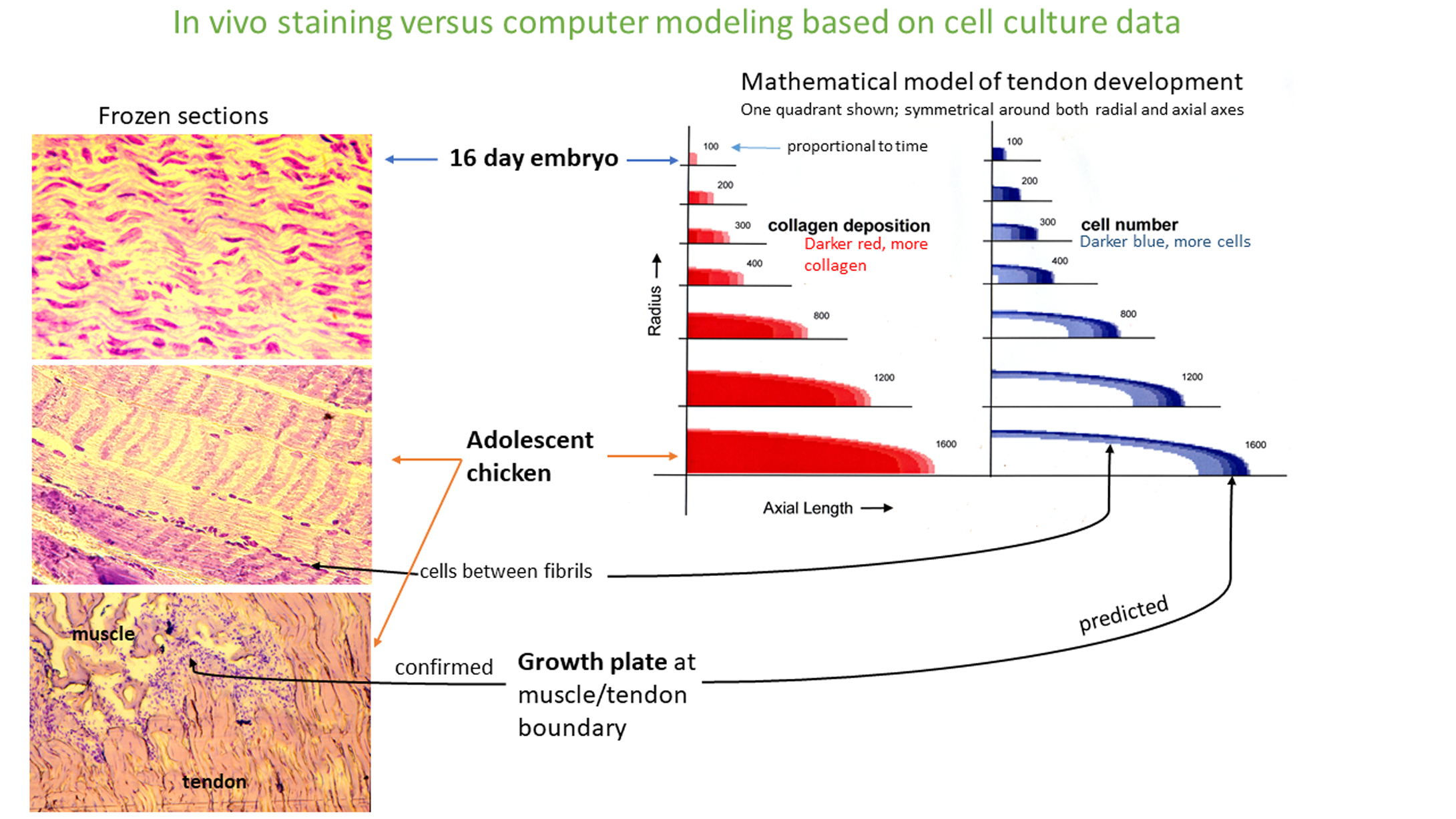

Supplement: Supplemental Information 1 — Strong correlation between the major changes observed in the histology of the tendon as it goes from a 16 day embryonic tissue to an adolescent tissue is observed in the computer simulation using the 2-factor model. In the embryo the cells are present throughout the tissue at a moderately high cell density and are in a moderately loose collagen matrix. In the adolescent tissue the collagen matrix is dense and the cells are located between the fibrils (the histology was done on frozen tissue and this can disrupt some features; in this case, the tendon cells can become rearranged around the collagen fibril). The computer solution to three partial differential equations based on our 2-factor model show a simulation of tendon morphogenesis over time. This is a dynamic model so it is constantly changing with time. Collagen deposition is shown in red with deeper red being a more dense collagen matrix. Cell number is shown in blue with higher cell density being a deeper blue. The model predicts that the tendon is growing by using a growth plate. This was confirmed by histology sections at the muscle\tendon boundary. The model only shows one quadrant since the calculations are symmetrical around both the radial and axial axes. [file peerj-10-14533-s001.png]

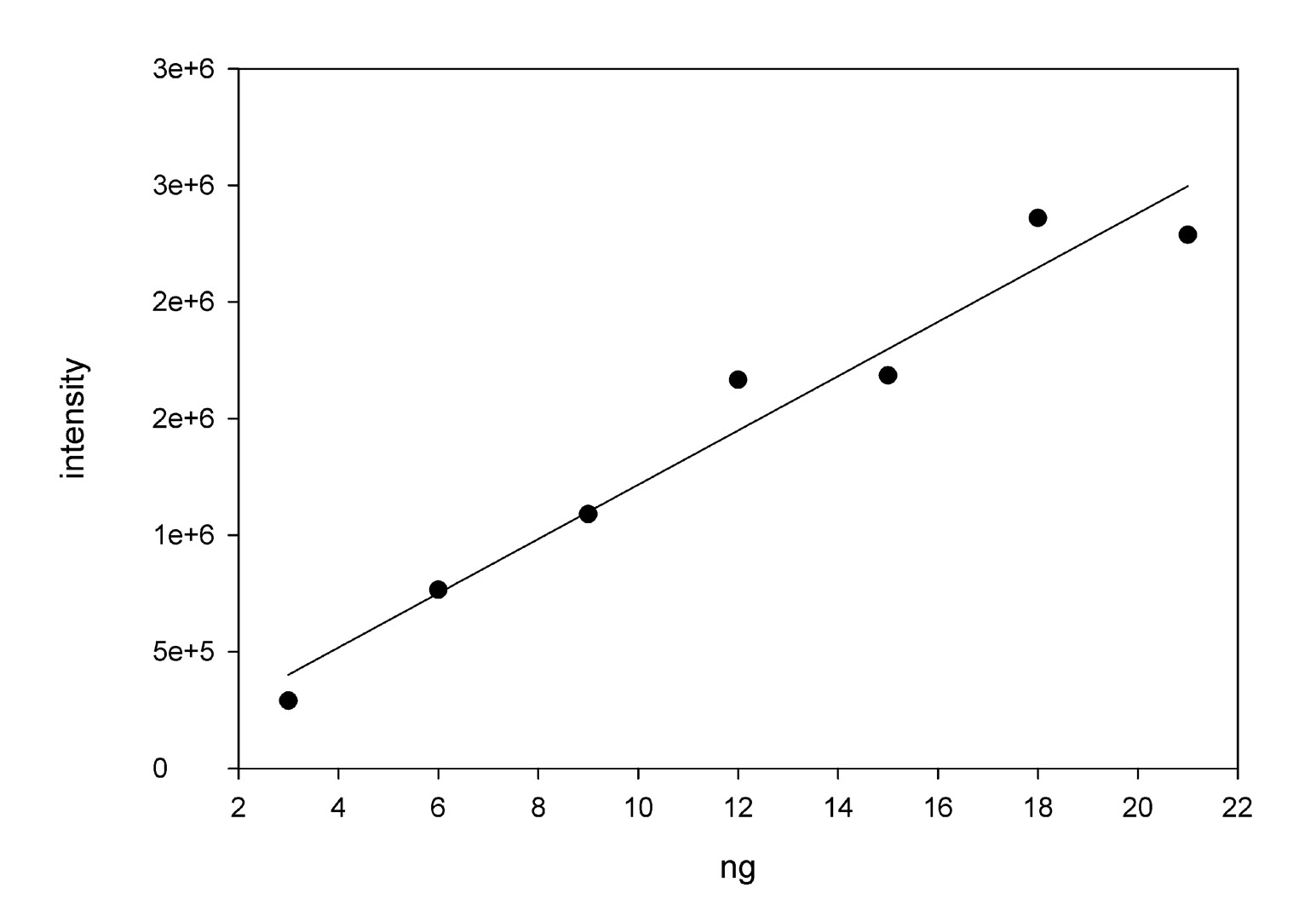

Supplement: Supplemental Information 2 — Various concentrations of RNAse A (3 ng to 21 ng) were run on an SDS gel and stained with Sypro Ruby. The methods used for running the gel and quantifying the staining were identical to those used to analyze the procollagen bands. This standard curve for intensity of the bands vs ng applied to the gel was used to convert the intensity of the procollagen bands to ng of protein. [file peerj-10-14533-s002.jpg]

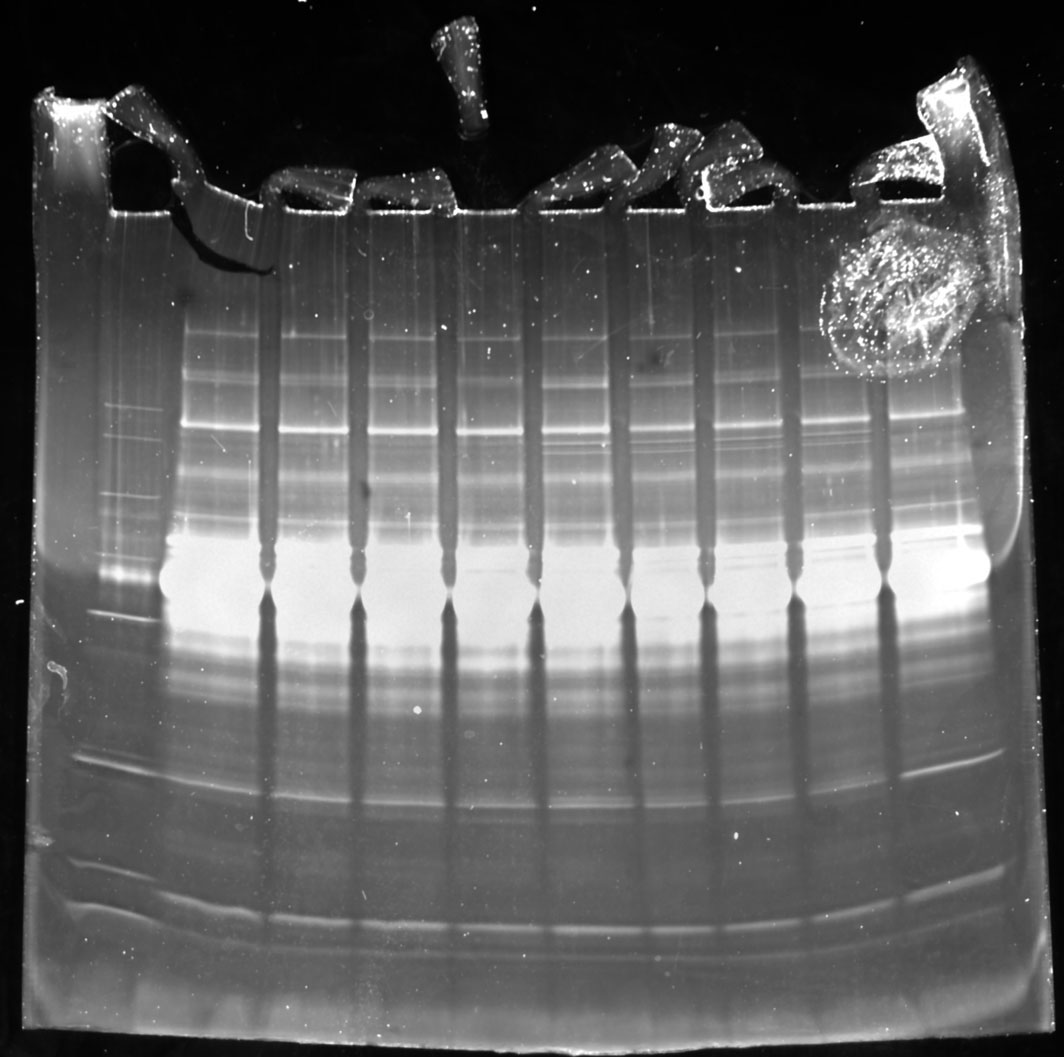

Supplement: Supplemental Information 3 [file peerj-10-14533-s003.jpg]

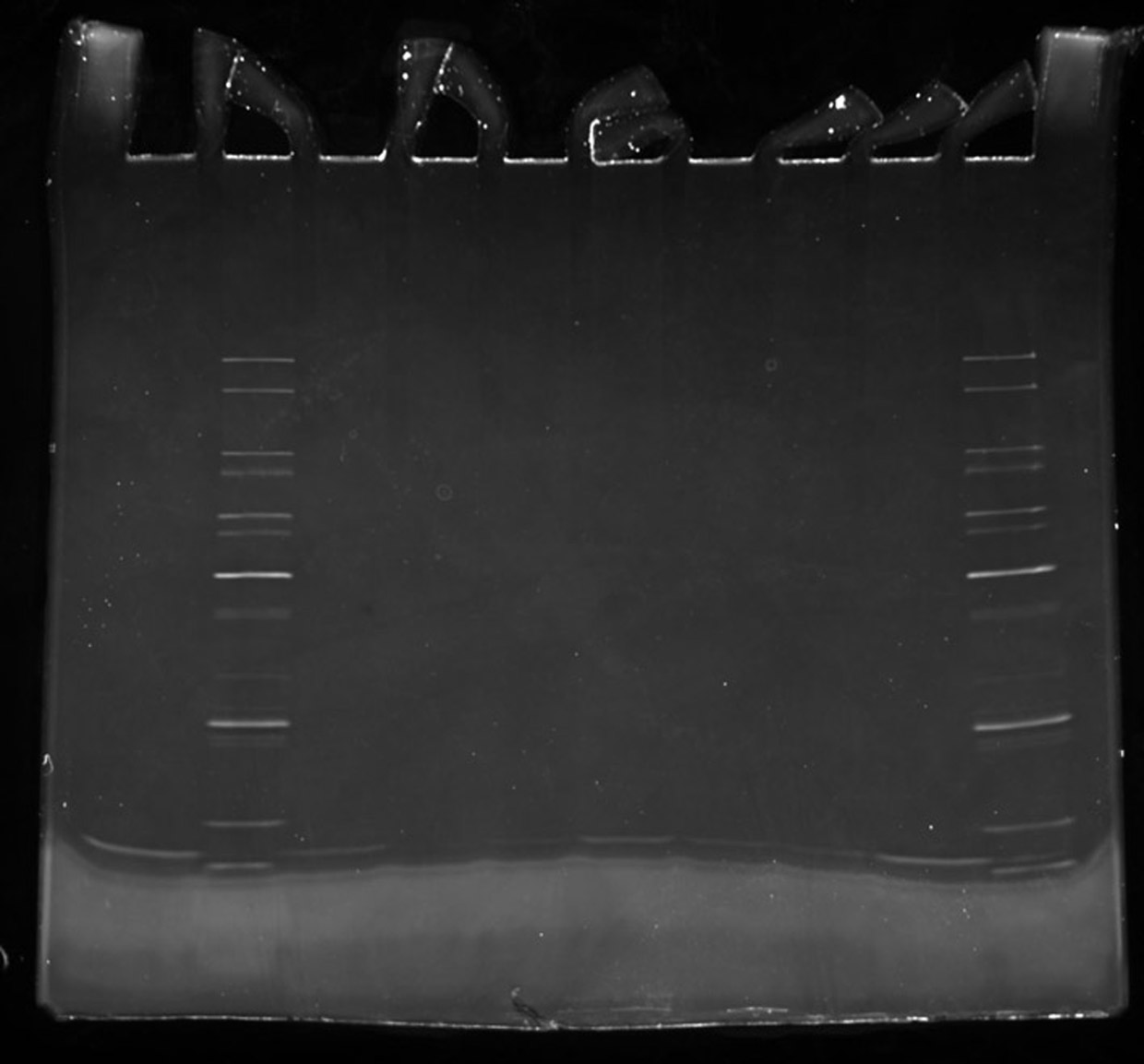

Supplement: Supplemental Information 4 [file peerj-10-14533-s004.jpg]
